# Design of an ankle exoskeleton with twisted string actuation for running assistance

**DOI:** 10.1017/wtc.2025.10010

**Published:** 2025-07-22

**Authors:** Guan Rong Tan, Steven H. Collins

**Affiliations:** Department of Mechanical Engineering, Stanford University, Stanford, CA, USA

**Keywords:** biomechanics, design, ankle exoskeletons, metabolic cost, mechatronics, running

## Abstract

Exoskeletons that make running easier could increase users’ physical activity levels and provide related health benefits. In this paper, we present the design of a portable, powered ankle exoskeleton that assists running and uses lightweight and compact twisted string actuators. It has limited durability at this stage of development, but preliminary results of its power to mass density and potential for reducing the metabolic cost of running are promising. The exoskeleton can provide high peak power of 700 W per leg, 7 times more than prior twisted-string devices, and high peak torques of 43 Nm. Kinetostatic and dynamic models were used to select mass-optimal components, producing a device that weighs 1.8 kg per leg and 2.0 kg in a backpack. We performed preliminary tests on a single participant to evaluate the exoskeleton performance during both treadmill running and outdoor running. The exoskeleton reduced metabolic energy use by 10.8% during treadmill running tests and reduced cost of transport by 7.7% during outdoor running tests compared to running without the device. Unfortunately, the twisted string wore out quickly, lasting an average of 4 min 50 s before breaking. This exoskeleton shows promise for making running easier if string life challenges can be addressed.

## 1. Introduction

Engagement in physical activity such as running has been strongly correlated to many health benefits. It can improve cardiovascular health (Shiroma and Lee, 2010; Piercy and Troiano, 2018), reduce symptoms of depression and anxiety (Stathopoulou et al., 2006; Mikkelsen et al., 2017), and reduce risk factors for a wide range of chronic diseases (Shiroma and Lee, 2010; Warburton et al., 2010). Increasing one’s perceived ability, enjoyment, and interest in running, while reducing exertion and activity intensity have been shown to lead to increased frequency of exercise and energy expenditure (Frederick and Ryan, 1993; Zunft et al., 1999; Dishman et al., 2005). A similar effect has been seen in cycling, where electric-assist bicycles have been shown to reduce barriers to physical activity and lead to an increase in weekly activity levels in non-cyclists (Castro et al., 2019). This suggests that exoskeletons that reduce the energy cost of running could play a role in encouraging greater participation in physical activities.

Various devices have been successful in reducing the metabolic cost of running. The Nike Vaporfly shoe with a carbon fiber plate embedded in the sole obtained a 4% reduction (Hoogkamer et al., 2018). Placing an elastic band between participants’ feet resulted in a 6% reduction (Simpson et al., 2019). Various hip exosuits and exoskeletons have also been designed. Two provided hip flexion assistance through a passive elastic element and achieved a 5% (Yang et al., 2021) and 6% (Zhou et al., 2021) reduction; one exosuit provided powered hip extension assistance and obtained a 4% reduction (Kim et al., 2019), and one exosuit placed a passive torsional spring at the hip that provided both hip flexion and extension torques and obtained an 8% reduction (Nasiri et al., 2018). These devices have enabled modest metabolic cost reductions. However, providing assistance to different muscles or using more power-dense transmission systems could make it possible to achieve even larger reductions.

Providing ankle plantarflexion assistance during running can result in large reductions in metabolic energy. Ankle plantarflexion provides the largest share of power during running (Novacheck, 1995) compared to other joints and actuation directions such as knee and hip flexion and extension. This means that for exoskeletons that assist a single joint, offloading the ankle plantarflexors could lead to the largest metabolic benefit. This has been shown to be true for walking (Franks et al., 2021), which is an activity that also receives the largest power input from ankle plantarflexors. A tethered device that provided powered ankle plantarflexion assistance during running achieved a 15% reduction in metabolic cost compared to running without the device (Witte et al., 2020). This suggests that we could transfer these large benefits to a portable device if we design it in a mass-optimal way.

A portable device for reducing the metabolic cost of running needs to have a high power-to-mass ratio, as high peak torques correlate to larger metabolic savings (Miller et al., 2022), and a heavier worn mass results in greater metabolic costs (Martin, 1985; Myers and Steudel, 1985; Hoogkamer et al., 2016; Coifman et al., 2023). Sufficient gearing is also necessary as electric motors can easily match the power of muscles, but operate at much higher speeds and lower torques.

Twisted string actuators are a less explored type of transmission for low limb exoskeletons and could provide the desired high power and gearing in a lightweight and compact package. We expect them to be much more lightweight than other gearbox solutions. Twisted string actuators work by having a set of strings attached coaxially to the motor shaft on one end and to the load on the other end. When the motor shaft spins, the strings twist together and shorten, generating a contraction force. The strings function as almost massless gearboxes while achieving high transmission ratios and a conversion from rotary to linear motion with minimal mechanical complexity. Twisted string actuators have been used in previous upper-body exoskeletons for providing elbow and shoulder assistance (Popov et al., 2013; Gaponov et al., 2016; Hosseini et al., 2020), as well as in lower limb exoskeletons such as a knee exosuit that provides stair climbing assistance (Zhao et al., 2019) and a hip exoskeleton that assists with lifting tasks (Seong et al., 2020). Various models of the actuator have also been developed. There are kinetostatic models of single string (Gaponov et al., 2014) and multiple string (Palli et al., 2013) applications and a dynamic model that accounts for effects of friction and a more complex elastic deformation effect (Nedelchev et al., 2020). We can use these models to perform inverse modeling (Bombara et al., 2022) to select the most lightweight motor that can perform our required output task.

In this paper, we describe the design of a portable, powered ankle exoskeleton for providing running assistance. The unique contributions in this work are as follows:

1. We present the preliminary design of the first exoskeleton that provides running assistance using twisted string actuators.
2. We demonstrate the highest power application of twisted string actuators to date. We reach peak powers of 700 W and mean powers of 49 W, which are 7 times and 4.5 times that of previous devices. Our device has a limited durability of 4 min 50 s when operating under such conditions.
3. We obtained large metabolic savings of 10.8% compared to running in normal shoes in a preliminary evaluation on a single subject.

We will give an overview of the device, discuss how we modeled the transmission to select lightweight device components, discuss the design of the device, and demonstrate the device’s ability to provide running assistance in both indoor and outdoor tests. We then discuss the benefits and challenges involved in achieving this outcome and future work.

## 2. Device overview

**Figure 1.**
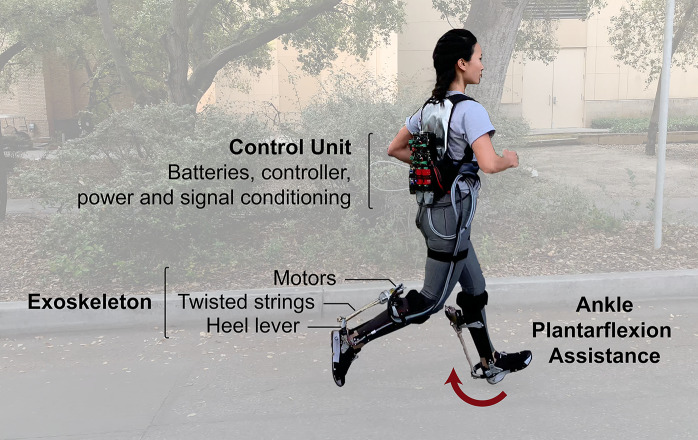
Overview of system components. The user wears an ankle exoskeleton around the shank and foot that applies ankle plantarflexion assistance torques. A control unit is carried on the user’s back.

We designed a portable ankle exoskeleton that provides running assistance (Figure 1). Motors are located behind the calf, and a set of strings attaches the motor shaft to a heel lever. Twisting the strings causes contraction of the string assembly, pulling the heel lever up and generating an ankle plantarflexion torque. Sensors in the device allow us to provide control feedback for tracking a desired torque profile that was determined in a previous study (Miller et al., 2022). The user also carries a control unit on their back which houses batteries, power and signal conditioning components, and control hardware.

## 3. Modeling and selection of transmission components

We modeled the twisted string kinetostatics and system dynamics to select the minimal mass motor and string combination that could accomplish our desired output task.

**Figure 2.**
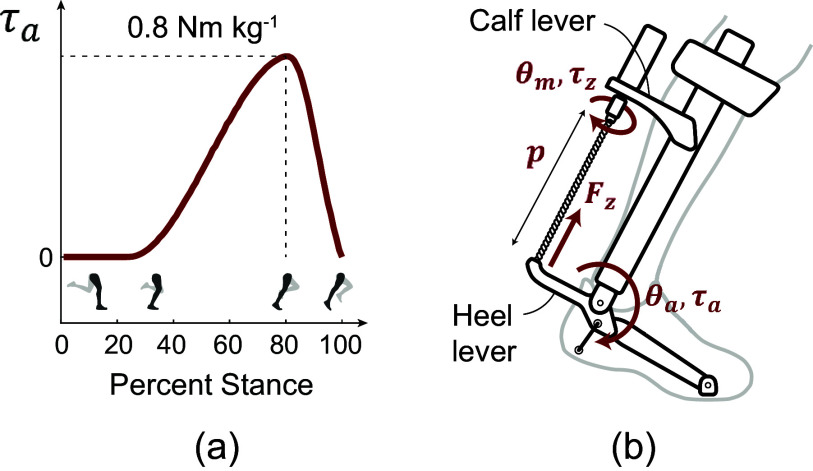
Exoskeleton torque application (a) Desired ankle torque assistance with peak torques of 0.8 Nm kg 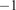 normalized to the subject’s body mass. (b) Schematic showing how ankle torque assistance is generated by pulling on the heel lever via contraction of the twisted strings. Key parameters and variables are described in the main text.

The output task was obtained from a previous study. The study performed human-in-the-loop optimization to identify a plantarflexion torque assistance profile that is optimal for reducing the metabolic cost of running (Miller et al., 2022). We aimed to replicate this torque assistance profile 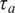 , shown in Figure 2(a) which had a peak torque of 0.8 Nm kg 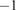 , normalized to the subject’s mass, that occurred at 80% of stance. We also extracted the corresponding ankle position trajectory 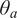 during torque assistance from the same study.

Based on device geometry, we can then calculate the twisted length 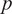 and axial force 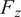 that the twisted strings need to apply to the heel lever (Figure 2(b)) in order to produce the desired plantarflexion torques 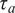 at an ankle angle 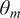 . Both 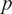 and 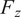 can then be fed into the kinetostatic model of twisted strings described in Section 3.1 to derive 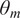 , the angular displacement of the motor and twisted strings, and 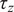 the axial torque applied to the twisted strings. We then model the transmission dynamics to obtain the motor operating points in Section 3.2 and describe how we iteratively find the final minimal mass motor and string combination in Section 3.3.

### 3.1. Twisted string kinetostatic model

The kinetostatic model of twisted string actuators describes how the rotational torque and displacement of the motor translate to the linear force and displacement of the twisted strings. This model has been discussed in detail in previous papers (Palli et al., 2013). Here, we present a condensed version to convey the key elements. In our design, we opted to use two strings for simplicity, and most of the equations apply specifically to this choice. Our model accounts for elasticity in each string:(1)

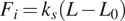

Where 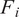 is the tensile force through each string, 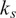 is the string’s spring constant, 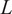 is the stretched length, and 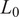 is the initial unstretched length.

**Figure 3.**
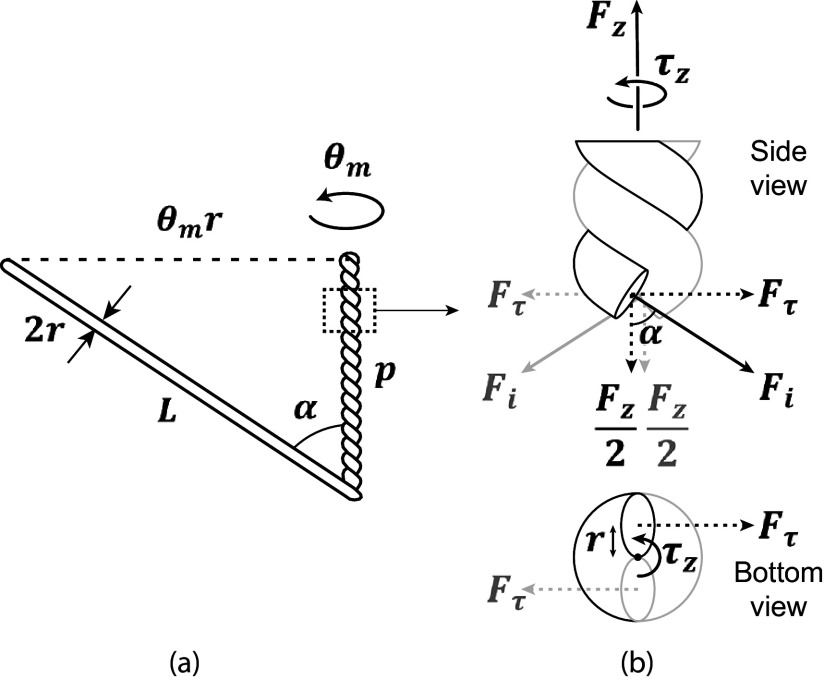
Kinetostatic model of twisted string actuators. (a) Geometric relationship between a string’s twisted length 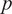 , untwisted length 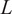 , twist angle 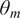 , radius 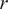 , and helix angle 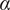 . (b) Free body diagrams demonstrating the force transmission through twisted strings. Strings are assumed to be massless, and diagrams are in static equilibrium.

Figure 3(a) demonstrates some key geometric relationships in a twisted helix. When two strings are twisted together, the untwisted length 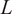 is related to its twist angle 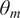 , radius 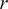 _,_ and twisted length 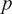 by the Pythagorean theorem such that(2)

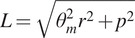

The relationship between the previous variables is also defined by the helix angle 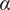 . The same helix angle defines how the tensile force through each string, 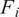 , shown in Figure 3(b) can be split into its two components, actuation force 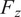 , and torsional force 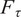 . Since the triangle formed by the dimensional parameters 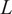 , 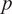 , and 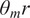 and the triangle formed by the forces 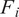 , 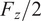 , and 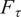 are similar, we can get the following two relationships:(3)

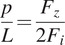


(4)

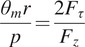



By substituting (1) and (2) into (3), we get(5)

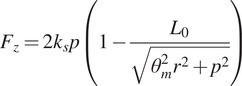



Rearranging (5), we can obtain motor angle 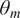 as a function of string parameters 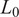 , 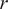 , 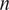 , 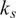 and output task requirements 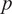 and 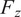 .(6)

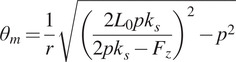



We also know from Figure 3(b) that(7)

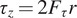



By substituting 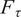 from (4) into (7), we get(8)

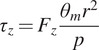



We can now use 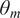 and 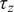 to calculate the motor operating point in the next section.

### 3.2. System dynamics

**Figure 4.**
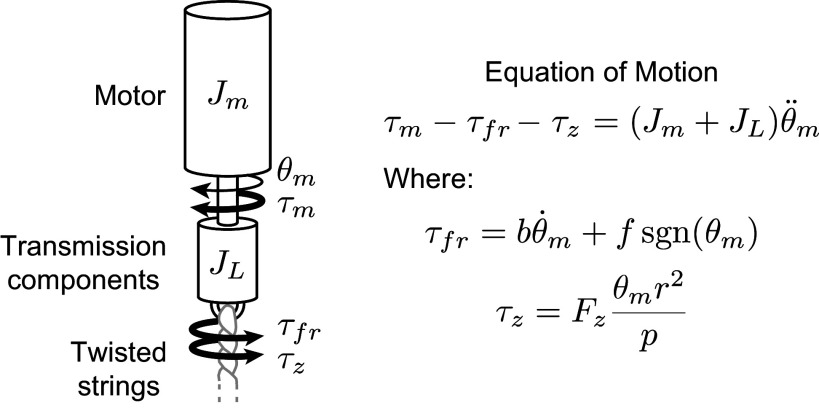
Simplified model of transmission dynamics.


We created a simple dynamic model of the transmission to find the motor torque 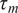 required to provide the desired 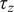 and 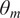 trajectory. The transmission components connecting the motor to the twisted string are described in more detail in the device design Section 4.3, but in this section, we modeled them as a single component with equivalent rotational inertia 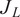 . The simplified model and resulting equation of motion for finding 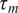 are shown in Figure 4.

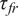
 represents the torque due to friction in the transmission. We modeled viscous and Coulomb friction in the motor and transmission bearings with 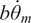 and 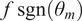 . Axial torque from the twisted strings 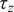 is derived in equation (8). 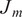 and 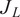 are the rotational inertias of the motor and transmission components, respectively. We can obtain 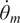 and 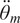 by differentiating 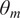 in equation (6).

A suitable motor for the task should be able to apply the desired 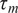 found at speeds 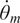 while staying within the following two motor limits. First, the motor should stay within its voltage saturation limit. This means that it must maintain 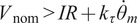 , where 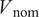 is the nominal voltage supplied to the motor, 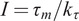 is the motor current, 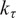 is the motor torque constant, 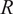 is the motor winding resistance, and 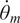 is the motor velocity. Second, the motor should stay below its thermal limits. It should maintain 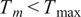 , where 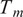 is the motor temperature and 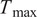 is temperature threshold of the motor windings from the datasheet. 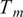 was calculated using the equation 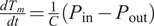 , where *C* is the motor thermal capacitance from the datasheet, 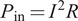 , and 
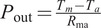
 where 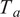 is the ambient temperature and 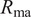 is the motor to ambient thermal resistance. 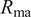 was calculated based on baseline values listed in the datasheet and effects from the additional airflow expected during outdoor running. The steady-state motor temperature trajectory for each given 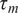 trajectory must not exceed 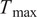 . By building our own thermal model, we could estimate motor thermal performance for our specific duty cycle and ambient cooling effects.

String parameters such as string radius, initial length, and stiffness affect the power requirements and transmission ratio, which, in turn, affect the ability of a motor to fulfil the aforementioned requirements. To handle this, we used an iterative selection process that we describe in the next section to find the best motor and string combination.

### 3.3. Iterative selection of motor and string

We systematically searched through the Maxon motor catalog to find the most lightweight motor and string combination for our device.

The motor had to be able to operate at the desired 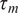 and 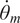 without exceeding voltage and thermal limits, and the string parameters had to satisfy the following design constraints:

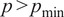

Where 
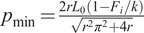
 is the shortest twisted length that a two-string bundle can contract to before buckling (Palli et al., 2013).

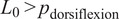

Where 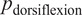 is the distance between the heel and calf lever during the subject’s most dorsiflexed ankle position during running.

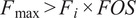

Where 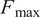 is the maximum tensile load rating of the string. FOS is the factor of safety used to account for string weakening due to knots and the sharp bending radius involved in the twisting.

We built our initial device based on the motor and string combination selected by the model. However, we found that the device was unable to provide sufficient power to track the desired torque profile. We found two contributing factors that explain why the initial selection did not work. First, there were key differences between the subject that ran in the exoskeleton and the subject whose ankle angle and torque data were used in the model. The subject that ran in the exoskeleton had an ankle range of motion that was 1.6 times what was input in the model. Since the stance to stride duty cycle of the subjects were similar, the device would require roughly 1.6 times the mean power to track the same torque profile across the wider range of motion. Since we were optimizing our design for having minimal mass, we chose the smallest motor possible that could accomplish the task, which meant that it wasn’t able to provide the additional power needed. To minimize mass while meeting power requirements in future designs, specifications for the power train should be chosen based on subject-specific gait parameters, with a variety of sizes available. Secondly, we had underestimated the friction losses across the twisted strings. The final device had an efficiency of 79% across the twisted strings. This should be taken into account when modeling twisted string actuators in the future. We rebuilt the device with two motors on each leg to increase its power output due to the aforementioned concerns. Dividing the torque across two pairs of twisted strings also helped to decrease wear on the ropes and increase rope lifespan, which was a major challenge that we described in more detail in Sections 5.5 and 7. We present this final design in the following section.

## 4. Device design

In this section, we present the design of the device’s mechanical components, sensor setup, and controller.

### 4.1. Component distribution

Mass worn more distally from a person’s center of mass incurs larger metabolic costs when running. Every additional 100 g on each ankle costs around 1% (Martin 1985; Hoogkamer et al. 2016; Coifman et al., 2023) versus 0.8% if worn on the shank (Coifman et al., 2023) and 0.1% if worn at the hip (Myers and Steudel, 1985). Due to this distribution, some existing twisted string devices were designed to have their actuators on the user’s back and transferred mechanical power to the limb through cable drive systems (Gaponov et al., 2016; Zhao et al., 2019; Hosseini et al., 2020; Seong et al., 2020). However, this results in additional complexity, energy losses through the cable drive systems due to friction, and a reduction of control bandwidth due to the additional compliance. To balance the two competing concerns of mass and complexity, we placed our actuation components on the leg and kept the remaining components such as the batteries and control hardware close to the user’s hips.

### 4.2. Sensors

**Figure 5.**
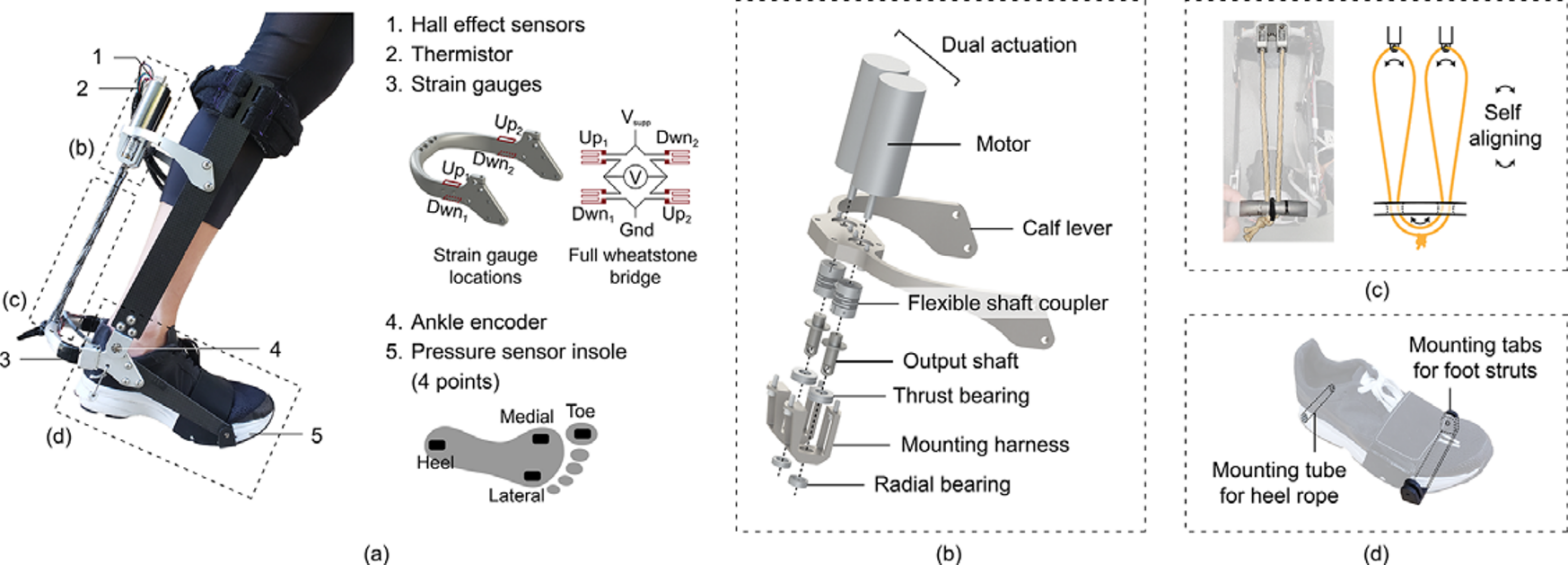
Sensors and mechanical components of the exoskeleton. (a) Sensor locations on the exoskeleton. Boxed-out components on the exoskeleton are detailed in Figure 5(b–d). (b) Exploded view of transmission components. (c) Routing pattern of the rope that allows self-alignment during torque application to equalize the lengths of the rope between the motor and the calf lever. (d) Mounting hardware incorporated in the shoe to allow exoskeleton attachment.

**Figure 6.**
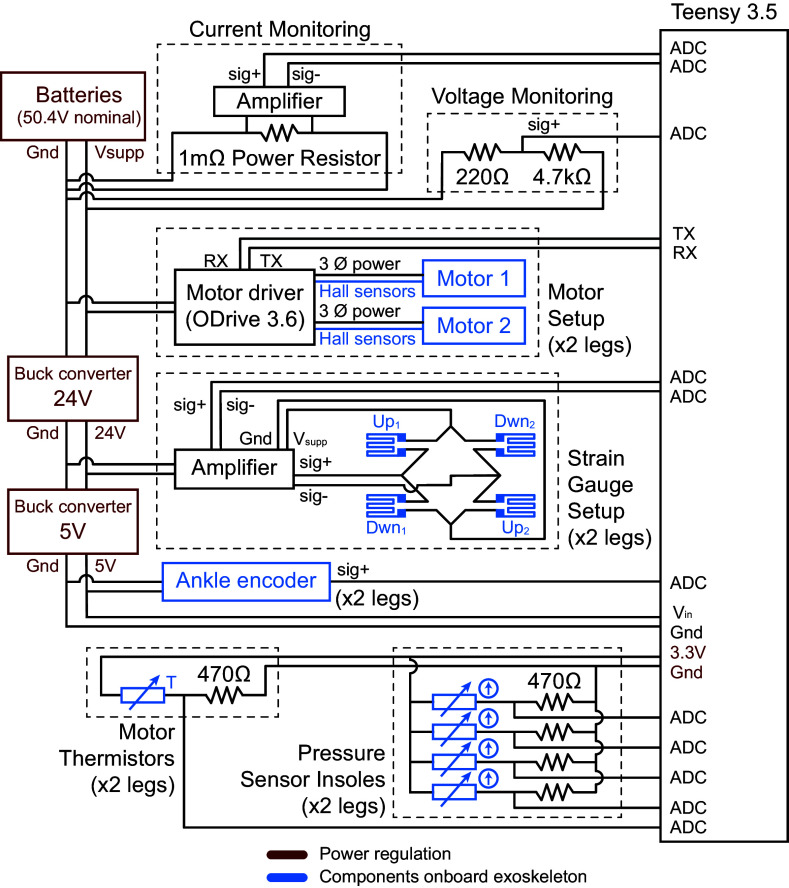
Schematic of power flow and sensor wiring distributed across the exoskeleton and control unit backpack.

A set of sensors on the exoskeleton shown in Figure 5(a) provided important state feedback that allowed us to implement an effective controller. The wiring diagram of the sensors is shown in Figure 6.

#### 4.2.1. Hall effect sensors

Hall effect sensors in the motor provided us with motor position feedback. They were wired directly into a motor driver (ODrive 3.6) that processed the signals and passed readings to our microcontroller (Teensy 3.5).

#### 4.2.2. Thermistors

Thermistors were placed on the surface of the motors to estimate motor temperature. This allowed us to operate in the noncontinuous operating zone of the motor to maximize torque output without overheating.

#### 4.2.3. Strain gauges

Strain gauges were used to measure the ankle torque assistance applied by the exoskeleton. Four strain gauges (Omega linear gauge KFH-3-350-C1-11L1M2R) were attached to the heel lever. Contraction of the twisted strings caused compression in the upper strain gauges and extension in the lower strain gauges. They were wired in a full Wheatstone bridge configuration, and signals were amplified by an instrumented amplifier (Futek IAA100).

#### 4.2.4. Ankle encoder

Magnetic encoders were placed at the ankle joint to measure ankle angle. We used this to estimate work done on the user and implement safety cutoff of torques if users got close to the limits of their range of motion.

#### 4.2.5. Pressure sensor insole

We used an insole that had four pressure sensor pads located under the heel, the toe, the medial edge under the first metatarsophalangeal joint, and the lateral edge under the fourth metatarsophalangeal joint. This was used to detect heel-strike and toe-off events for determining gait cycle progression.

### 4.3. Transmission

The transmission interface shown in Figure 5(b) mechanically connects the motors to the twisted strings. The motor body is mounted above the calf lever, and a flexible shaft coupler connects the motor shaft to a stainless steel output shaft below the calf lever. The output shaft has an eyelet on one end through which the twisted strings are looped.

Motors are designed to transmit large torques through their motor shafts, but the shafts can only sustain minimal axial and radial forces. To provide protection against such forces, the output shaft is supported by a radial bearing and has a flange that braces against a thrust bearing. These bearings are attached to the mounting harness that is bolted to the calf lever. The bearings helped ensure that the large axial forces generated by the twisted string contraction and the radial forces generated from the non-colinearity between the twisted strings and the motor axis were not passed on to the motor shaft. These bearings also helped reduce energy lost to heat generation through sliding friction. All components interfacing with the radial bearings were toleranced for a locational clearance fit to improve alignment.

Free body diagrams and finite element analysis were used to design components with minimal mass and rotational inertia that could sustain the expected dynamic loading.

### 4.4. String setup

A single 3 mm-diameter single-braid V-12 Vectran (West Marine, Model #155791) rope was used across each dual motor setup per leg and was routed as shown in Figure 5(c). This routing pattern allowed the ropes to slide through the output shaft eyelets and guiding holes in the calf lever as shown by the arrows. During torque application, this movement would cause the rope lengths between each motor and the calf lever to equalize, leading to even loading across the two motors. The ends were tied off with a three-throw square knot. This knot selection was favorable as additional resistance against slipping or loosening could be added easily by adding additional throws. Lubricating grease (Krytox GPL 205) was applied to the inside of the braid evenly along the entire length of the Vectran by a curved tip syringe and massaged into the fibers. This helped to reduce and distribute the thermal load on the Vectran caused by friction during twisting.

### 4.5. Structural frame

The exoskeleton frame comprised carbon fiber struts and custom aluminum and stainless steel components. It was a modification from a previous design described in more detail in Witte and Collins (2020). The frame was designed such that the exoskeleton would be comfortable for the user even at high torque assistance levels. The exoskeleton achieves this by applying normal forces to the user’s heel and tibia, rather than shear forces which are much less comfortable (Yandell et al., 2017). In this version, the medial-lateral device envelope was reduced to make it more suitable for running. The shoes also had new mounting hardware built into the sole as shown in Figure 5(d). A 3D-printed carbon fiber component sat between the layers in the sole and extended out from under the metatarsophalangeal joints to provide rigid mounting tabs for the exoskeleton hardware to interface with. A plastic mounting tube in the heel also protected the soft foam from abrasion by the heel rope that exerts large upward forces on the shoe.

### 4.6. Control unit

The user carries a control unit on their back that houses four lithium polymer batteries connected in series (Turnigy Nano-Tech 3300 mAh 3 S 25 C), the microcontroller (Teensy 3.5), motor drivers (ODrive 3.6), voltage amplifiers (Futek IAA100), and voltage regulators (48 to 2 V, and 24 to 5 V) in a 3D-printed frame attached to a modified open face backpack. This brings the device mass closer to the user’s center of mass which minimizes the metabolic cost of carrying the additional weight.

### 4.7. Controller

Motors on the same leg received identical commands. However, each leg ran independent controllers since slight differences in string length and string wear between the two legs would cause variation in the desired control command. A gait phase detection algorithm used pressure sensor insole readings to detect stance and swing phases by detecting heel-strike and toe-off events. Duration estimations of the stance and swing phases of each new step was calculated based on a moving average of the previous 5 steps. This value was carefully selected; 5 steps were few enough to allow the exoskeleton to be sufficiently responsive to the natural variations in the subject’s speed during outdoor running, while still enough steps to provide sufficient stability for the iterative learning component of the controller which we describe in the next paragraph. The controller operated in torque tracking mode when the user was in the stance phase and position tracking mode when the user was in swing phase.

During torque tracking mode, the controller tracked the desired torque commands shown in Figure 2(a) using a proportional term and iterative learning term. The iterative learning component, explained in Zhang et al. (2017a), is designed for cyclic tasks. Running can be seen as a cyclic task if separate strides are viewed as different cycles with the same desired torques. The iterative learning term leverages the repetitive cycles to compensate for systematic errors by using errors from previous steps to inform the desired command for the next step. This allows it to act as a feedforward term without the need for an explicit model. We commanded the desired velocity of the motors based on the following control law:

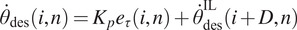

where *i* is the time index within a step, n represents the step number, 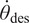 is the desired motor velocity commanded, 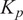 is the proportional gain, 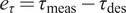 is the torque error from subtracting desired torque from torque measured by the strain gauges, and 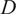 is the time delay between the command and the application of torque. 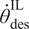 is the iterative learning term as shown below:

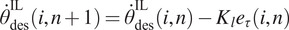



Both 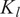 and the number of steps used to calculate the moving average stance time were tuned to ensure that the system was sufficiently stable during outdoor running, where the user experienced larger variations in their gait patterns. Desired torque profile was applied as a function of time, rather than gait percentage, as we could not know the stance time of the current step until it was over. This meant that if the number of steps used to calculate stance time was too few, or 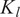 was too high, there would be high sensitivity to variations in stance time or gait pattern. We found that using a moving average of 5 steps to calculate stance time was the minimum number needed for the exoskeleton to provide sufficient stability, while low enough to allow the exoskeleton to be sufficiently responsive to the natural variations in the subject’s speed during outdoor running. 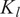 was also tuned in tandem to be large enough to respond to any changes in general gait patterns, without being too sensitive to step-to-step variations due to uneven or unpredictable terrain. In the next section, we describe how we assessed that we had tuned the device to a sufficiently stable state to be safe and robust for outdoor running.

In swing, the controller switched to position control mode and unraveled the twisted strings to provide slack and allow the user to swing their ankles freely.

### 4.8. Safety

High-power portable running devices need to have carefully designed safety features. The peak torque of the device is around half the biological peak torque level seen in running (Novacheck, 1995), making it nontrivial for the subject to overpower the device. Having a highly robust system was also important as stumbling while running could lead to injury.

We ensured that our device could provide reliable and consistent assistance for outdoor usage with zero missteps or step-to-step torque variations of more than 25% for the duration of a 6-minute trial. This duration was selected to be comfortably longer than the expected trial time of any single condition during our outdoor running tests. It was possible to obtain 6 min of running data as the rope lifespan varied between trials. We repeated data collection trials until we obtained multiple trials where the ropes lasted longer than 6 min and evaluated reliability using only those tests.

Users experience a sudden drop in torque assistance when the ropes wear out. We took precautions to ensure that this would not result in any falls from device use. When running indoors, the subject wore a safety harness mounted to the ceiling to prevent them from falling. For outdoor tests, we ensured that running durations were comfortably below the expected rope lifespan.

The handheld controller was wired in a “deadman switch” configuration such that active pressure on a button under the user’s thumb was required to continue commanding assistance from the device. This ensured that commanded torques would be cutoff if the user let go of the controller in the case of a loss of control.

Ankle encoder data were also used to ensure that the exoskeleton didn’t overextend the user’s ankle. Each user’s comfortable ankle range of motion was recorded at the start of experiments, and the controller was prevented from commanding assistance beyond a comfortable threshold.

## 5. Device performance

We assessed the device’s torque tracking performance, battery life, power efficiency, mass, and string lifespan while applying assistance during running. These are key performance indicators of a portable assistance device.

Tests that involved a person in the device were performed on a single subject (female, 165 cm, 54 kg) at a running speed of 2.68 m s 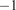 . This is equivalent to a 10-min mile pace, an approximation of the jogging speed of a recreational runner.

### 5.1. Torque tracking

**Figure 7.**
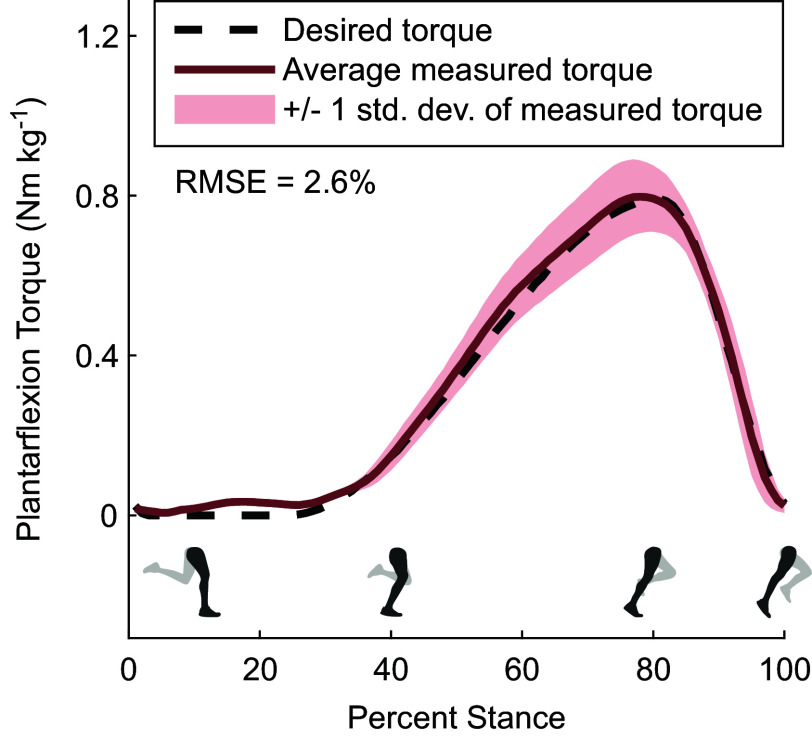
Torque tracking results.

We tested the device’s ability to track the desired torque profile shown in Figure 7 which had peak torques of 0.8 Nm kg 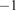 , which corresponded to 43 Nm for our subject, occurring at 80% of stance. We collected data on the measured torques over 20 steps after the exoskeleton controller had reached a steady state. The device accurately tracked the desired torque profile during the stance phase with a root-mean-square error of 0.021 Nm kg 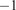 or 2.6% of peak torque.

### 5.2. Battery life

We monitored the current draw from the batteries by placing a 0.001-Ohm power resistor in series with the battery, passing the voltage difference through an amplifier, and reading the voltage into our microcontroller as shown in the “Current Monitoring” block of Figure 6.

**Figure 8.**
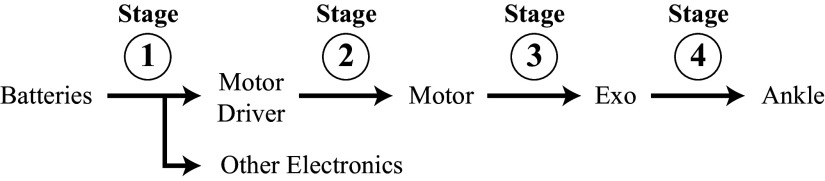
Device power flow diagram.

**Figure 9.**
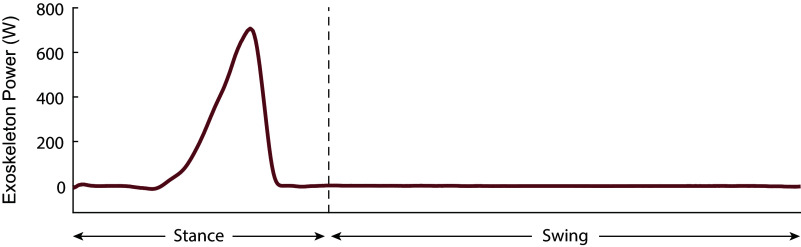
Exoskeleton power for one leg averaged across 20 strides.

**Figure 10.**
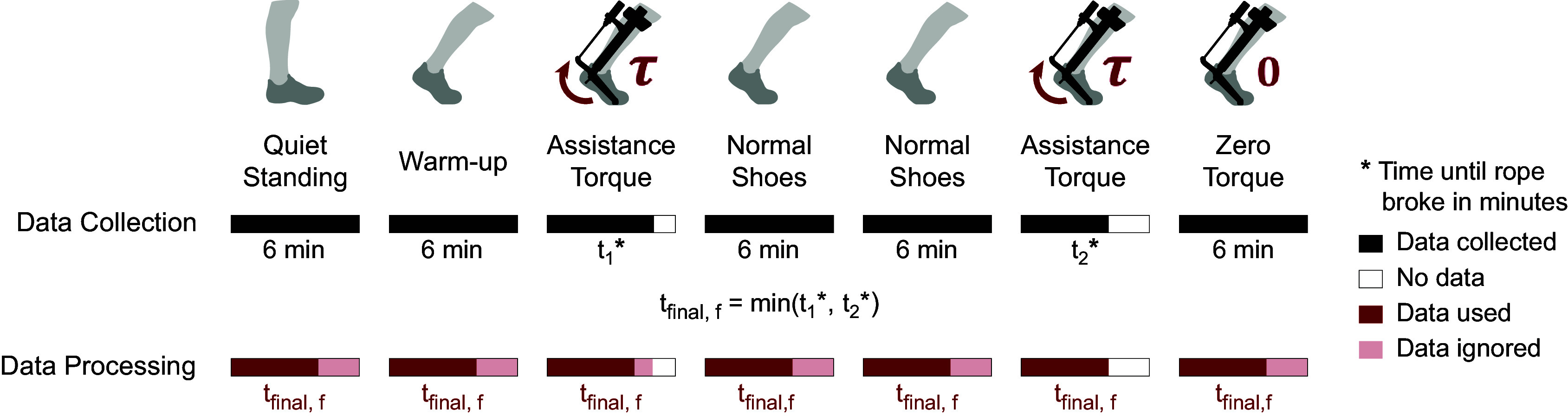
Experimental protocol of the fixed speed test. All conditions were conducted for 6 min except the assistance torque conditions which were conducted until the strings broke. 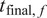 was set as the duration of the shorter assistance torque test. We then only used the first 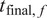 minutes of data from all conditions during data processing. This helped to reduce systematic bias in our metabolic results.

**Figure 11.**
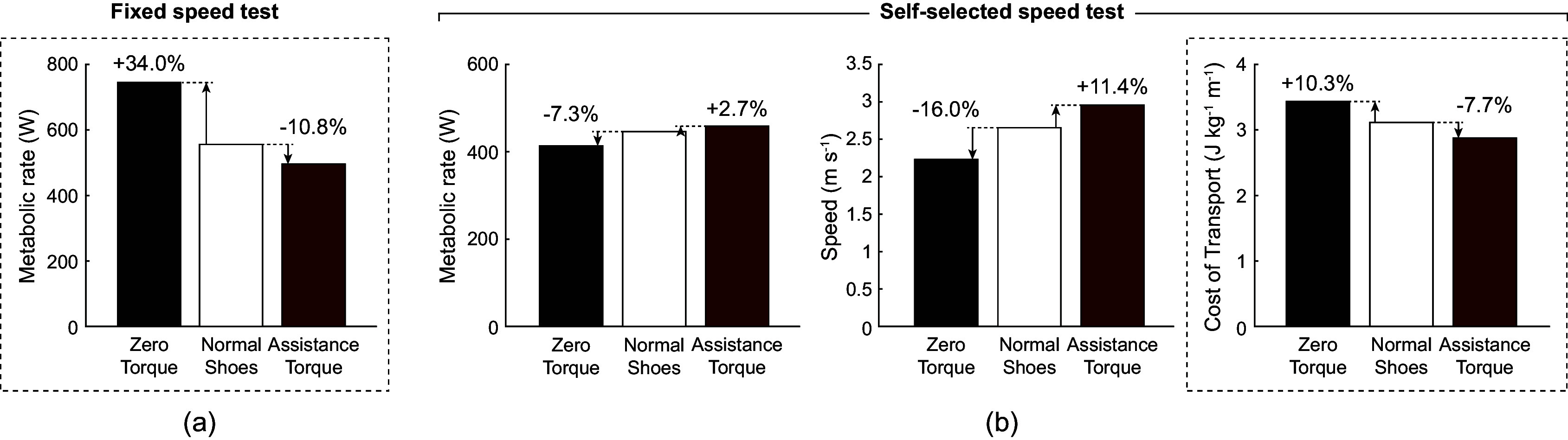
Results from the running assistance tests. Dotted lines indicate plots that are representative of the overall performance of each test. (a) Change in the subject’s metabolic rate over various conditions during the fixed speed test. (b) Change in the subject’s metabolic rate, speed, and cost of transport over various conditions during the self-selected speed test.

**Figure 12.**
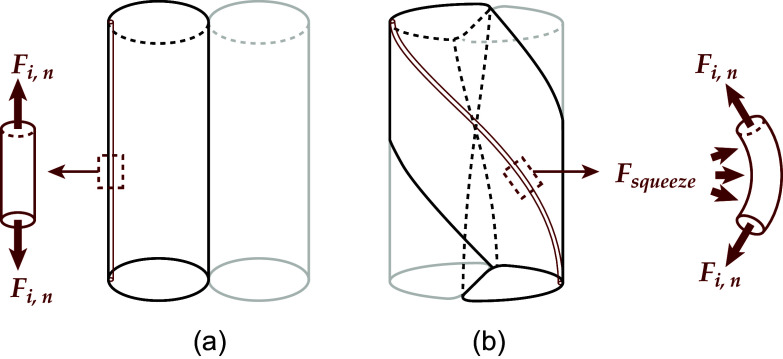
Intra-rope friction due to fiber rearrangement. (a) When ropes are untwisted, no squeezing force exists. (b) When ropes are twisted, tensile forces in the fibers 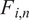 cause fibers to be pulled toward the twist axis. Static equilibrium is achieved when fibers apply a squeezing force 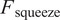 on those closer to the twist axis.

**Figure 13.**
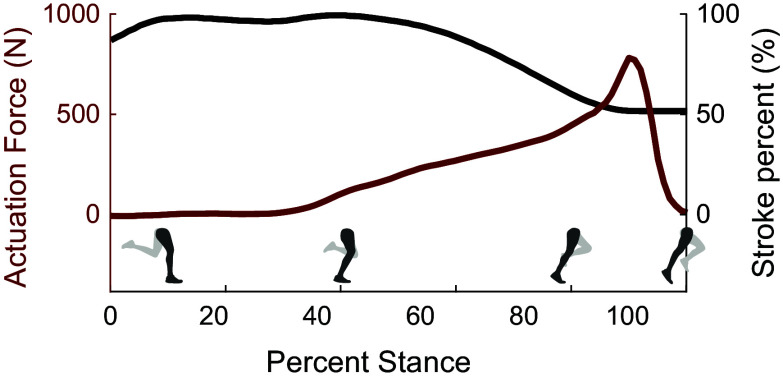
Actuation force 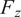 and stroke percentage 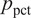 during stance.

During the battery life test, the subject ran with 43 Nm peak torque assistance. We only used trials where the rope lasted at least 5 min and collected data across the 5 min. The current draw was monitored over the duration that the controller was in steady state. To calculate the battery lifespan, we assumed that the batteries would be charged to their full capacity at 4.20 V per cell and used to 20% of their capacity or 3.75 V per cell in order to protect battery health and durability. We then calculated the theoretical battery life assuming the batteries experienced the measured steady-state current draw over its allowed voltage range.

The batteries would be able to support steady-state running with 43 Nm peak torque assistance for 28 min, corresponding to a run distance of 4.56 km at a 2.68 m s 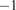 pace. The battery can be easily resized as needed to adjust the prioritization between device mass and run time.

### 5.3. Power efficiency

We measured the power output at four stages of the power flow to understand the efficiency of various components and sources of losses. The calculated average power, efficiency between stages, and overall efficiency of each stage over the first stage are shown in Figure 8 and Table 1. We have also included a plot of our device’s average power output across a stride in Figure 9.


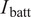
 and 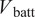 are the measured current and voltage output from the battery measured as shown in the “Current Monitoring” and “Voltage Monitoring” blocks in Figure 6. 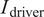 and 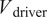 are the current and voltage output of the motor driver obtained from motor driver feedback. 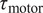 and 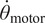 are the motor torque and motor angular velocity obtained from motor driver feedback. 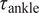 is the torque applied on the ankle obtained from strain gauge feedback and 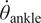 is the ankle angular velocity obtained from ankle encoder feedback.

The efficiency of the twisted strings alone is represented by stage 4 over stage 3 efficiency of 79%. This captures the difference between the power input from the motor into the strings and the power output from the strings into the exoskeleton. This efficiency is higher than other gearboxes that operate with a similar transmission ratio. For example, Maxon planetary and spur gearheads (Maxon, 2023) that allow transmission ratios of up to 250 like our transmission system have efficiencies of 60 to 75%. Harmonic drive gearboxes can reach efficiencies of 80 to 90%, but are much heavier and have lower transmission ratios.

Our device provided an average power of 49 W and peak power of 700 W to each ankle, which is much higher than the power levels provided by other similar devices. The next highest peak powers seen in running exoskeletons are 125 W in Kim et al. (2019) and 100 W in Nasiri et al. (2018). The next highest peak power seen in twisted string devices is 100 W in Seong et al. (2020).

These results were recorded during the first 2 min of running, while there was still a minimal amount of rope wear. We did not evaluate the impact of rope wear on power efficiency, but expect it to negatively impact results.

### 5.4. Device mass

The device mass is distributed across three areas: 0.7 kg around each ankle, 1.1 kg behind each calf, and 2.0 kg at the waist of the user. This sums to 1.8 kg on each leg and a total device mass of 5.6 kg. We discuss the reasoning behind selecting this mass distribution and the trade-offs considered in Section 4.1. Our device is heavier than previous running exoskeletons (Nasiri et al., 2018; Kim et al., 2019), but provides much higher average and peak power as described in Section 5.3.

Based on previously reported values on the metabolic cost of worn mass on the ankle, shank, and hips (Martin, 1985; Myers and Steudel, 1985; Hoogkamer et al., 2016; Coifman et al., 2023), the estimated metabolic cost of running with the device turned off is around 17%.

### 5.5. Twisted string lifespan

A major challenge we faced was the short lifespan of the twisted strings in our high-torque, high-speed application. We tested various high-performance ropes and noticed differing modes of failure between them. Kevlar, an aramid fiber rope, frayed and broke in under two minutes of testing. The fraying suggests that Kevlar has insufficient surface abrasion resistance. Dyneema, an ultra-high-molecular-weight polyethylene (UHMWPE) fiber rope hardened and split in under 3 min of testing. The hardened ends suggest that the rope exceeded its thermal ratings. Vectran, a liquid-crystal polymer fiber rope was the most robust and appeared to have a good balance between having surface abrasion resistance and a high thermal threshold. We used a 3mm-diameter single-braid V-12 Vectran rope (West Marine, Model #155791), rated to have an average breaking strength of 9.5kN and a 0.71% stretch at 20% breaking strength. We applied grease lubricant into the center of the braid and performed six running tests with peak torques of 43 Nm. The mean lifespan of the Vectran rope was 4 min 50 s.

An additional challenge presented by transmission wear is reduced efficiency and overall torque and power production. As the rope heats and wears, the interface between the twisting rope strands may have greater adhesion and larger surface features resisting sliding and conformation. In our experiments, we observed a reduction in peak torque and power in the push-off phase of the stance during the final strides before transmission failure (Supplementary Figure S1). This indicates that additional torque losses in the transmission saturated the motor drive, resulting in lower power applied to the ankle joint. This issue further motivates the development of more robust and efficient rope transmission configurations.

The limited string lifespan will be an even larger concern if higher torque levels are needed. The current lifespan of 4 min 50 s is achieved with the device commanding a torque profile with 43 Nm of peak torque, which corresponds to 0.8 Nm kg 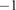 of torque for our subject weighing 54 kg. The body mass of a 50th percentile Caucasian male is 79 kg (Cassola et al., 2011). A subject weighing 79 kg would need peak torques of 63 Nm, almost a 50% increase from the 43 Nm tested in our experiments.

Thankfully, the lifespan we achieved was sufficient for us to perform the preliminary investigations mentioned in this paper and assess the device’s effectiveness in assisting running. Future design changes such as parallelizing actuation force through a higher number of twisted string pairs may increase rope life significantly without much increase in device mass and therefore have a minimal impact on the results of our running assistance tests. We further address methods to increase rope lifespan in Section 7.

## 6. Running assistance

We performed two preliminary tests on the device’s ability to make running easier. In the first “fixed speed test”, the subject ran at 2.68 m s 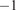 on a treadmill until the twisted strings wore out or for a maximum of 6 min, whichever came first, and we measured their change in metabolic rate when running with assistance torques. In the second “self-selected speed test”, the subject ran outdoors along an 815 m long path at a comfortable pace, and we measured both their change in metabolic rate and running speed in order to get estimates for their change in the cost of transport. We did not measure instantaneous running speed, but expect the effects of any possible speed fluctuations on the cost of transport to be small as has been observed for running in normal shoes (Kram and Taylor, 1990). These tests were performed on a single subject to evaluate the preliminary potential of the device. Additional tests on more subjects would need to be performed to ensure that results are generalizable. This was the same subject that ran in the device performance tests.

We applied the torque assistance profile as mentioned in previous sections with peak torques of 0.8 Nm kg 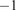 normalized to body weight for our subject, which corresponded to 43 Nm. We used a respirometry system (Cosmed, Quark CPET when indoors; Cosmed, K5 when outdoors) to measure oxygen consumption rates and carbon dioxide production rates in order to calculate the user’s metabolic rate. The subject also fasted for 3 h before the start of each session to minimize the thermic effect of food on metabolic rate measurements (Reed and Hill, 1996).

Previous tests showed that the twisted strings had a minimum lifespan of 3 min 22 s during 43 Nm peak torque assistance levels. A previous study Zhang et al. (2017b) showed that fitting an exponential curve to 3 min 22 s of metabolic data during a steady-state trial would produce a steady-state estimation with an error of 1.7%, while longer trials lead to further reductions in this error. The low error indicates that our trial lengths would be sufficient for getting accurate estimations of the steady-state metabolic rate.

### 6.1. Protocol

#### 6.1.1. Fixed speed test

The protocol for the fixed speed test is shown in Figure 10. The subject ran at a fixed treadmill speed of 2.68 m s 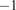 for all running conditions.

We first conducted the “Quiet Standing” condition, where we measured the subject’s baseline metabolic rate when standing at rest. We then conducted the “Warm-up” condition where the subject ran on the treadmill for 6 min in running shoes. Next, we performed the two main test conditions. For the “Assistance Torque” condition, the subject ran with the exoskeleton applying 0.8 Nm kg 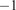 peak torques until the twisted strings wore out or for a maximum of 6 min, whichever came first. We did this instead of setting a trial duration below the Vectran rope’s minimum lifespan in order to maximize trial duration and minimize metabolic rate measurement error. Next, we performed the “Normal Shoes” condition where the subject ran wearing just the running shoes that the exoskeleton was attached to in order to keep shoe mass consistent. We performed a double reversal by applying a repeat of the “Normal Shoes” condition, then the “Assistance Torque” condition. This helped us account for the effects of subject fatigue on the metabolic rate measurements as the experiment progressed. We recharged the exoskeleton batteries and replaced the strings between the two “Assistance Torque” conditions. Last, we performed a “Zero Torque” test where the subject ran in the exoskeleton while it was turned off. This allowed us to understand the effects of the worn device mass on metabolic cost. We did this condition only once as it was heavily fatiguing and we did not want it to affect the subject’s response to the main test conditions of interest.

In order to minimize systematic bias when processing our metabolic cost results, we calculated 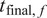 , the shorter of the two assistance torque condition durations, then used only the first 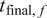 minutes of data from the rest of the conditions for calculating our final metabolic cost results.

#### 6.1.2. Self-selected speed test

The self-selected speed test had the same sequence of test conditions as the fixed speed test. However, the subject instead ran outdoors along a predetermined path. The path consisted mostly of paved sidewalks and included three 90-degree turns. The subject started and ended at the same points of this predetermined path for each condition, covering a fixed distance of 815 m each time. This allowed us to control for terrain and running surface. This distance was chosen so it could be comfortably completed before the twisted strings broke, allowing us to cover the same distance across all conditions. New strings were swapped in before each “Assistance Torque” condition. The subject was asked to run at a comfortable speed for all conditions, and we measured their run time, as well as their oxygen consumption rate and carbon dioxide production rate.

In order to minimize systematic bias when processing our metabolic cost results, we calculated 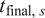 , the duration of the condition with the shortest run time, then used only the first 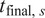 minutes of data from the rest of the conditions for calculating our final metabolic cost results.

### 6.2. Results

We calculated the metabolic rate from the measured oxygen consumption rate 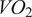 and carbon dioxide production rate 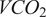 , based on a standardized equation: 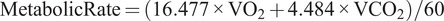 (Brockway, 1987). We then fit an exponential curve to the first 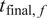 or 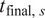 min of data for each condition to obtain the estimation of the steady-state metabolic rate. The final metabolic rate reported is then obtained by subtracting the metabolic rate of quiet standing from each condition. For the assistance torque and normal shoe conditions, we averaged the results of the repeated trials.

For the self-selected speed tests, speed was calculated by dividing the path distance of 815 m by the time taken to complete each condition. The cost of transport was calculated by dividing each condition’s final metabolic rate by the speed and the subject’s mass to get a mass-normalized cost of transport:(9)

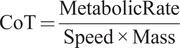



#### 6.2.1. Fixed speed test

The twisted strings lasted 5 min 30 s during the first assistance torque trial and 4 min 30 s during the second, leading to a 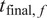 time of 4 min 30 s.

The results are shown in Figure 11(a). Running with assistance torque reduced the metabolic cost of running by 10.8%, while running in the zero torque condition increased the metabolic cost of running by 34.0%, both as compared to running with normal shoes.

The 10.8% reduction in metabolic cost when running with assistance torques is very promising. As a comparison, it is higher than the current best-performing device which produces an 8.0% reduction with a passive hip exoskeleton (Nasiri et al., 2018). Our tests only involved one subject, and these results suggest that we should perform tests with a larger group of participants to estimate the mean and variance of this benefit across the population.

The 34.0% metabolic cost increase in the zero torque condition is much larger than the expected 17% estimate based on the device mass distribution. While this outcome is not the central focus of the present study, it is worth speculating as to the possible reasons for the observed discrepancy. The standardized equation Brockway (1987) used for calculating metabolic rate is designed for calculating a person’s metabolic rate during aerobic respiration, but the subject had a respiratory quotient of greater than 1.0 for the final minute of the trial, suggesting that they were in anaerobic respiration. This could result in inaccuracies in the estimated metabolic rate. The subject did keep their respiratory quotient below 1.0 during the zero torque condition of the self-selected speed test, so we believe that the results from the zero torque condition produced in that test would be more reliable.

When comparing the device power input into the subject to the metabolic power savings achieved, we find that there is an amplification effect on the energy savings. The average power input into the subject of 98 W (stage 4 of Table 1 × 2 legs) is smaller than the total metabolic savings of 249 W between running with zero torque and running with assistance torques. This is lower than the savings that would be expected based on the conversion efficiency of skeletal muscle (about 25% Barclay, 2019) consistent with prior studies of the apparent efficiency of muscles under exoskeleton assistance (Sawicki and Ferris, 2009).

**Table 1.** Measurements of power and efficiencies of each stage over the previous stage and overall efficiency of the current stage over stage 1

Stage		Power (one leg)	Efficiency over the previous stage	Overall efficiency
1	Battery output 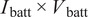	116 W	NA	100%
2	Motor driver output 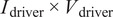	98 W	85%	85%
3	Motor output 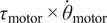	62 W	63%	53%
4	Exo output 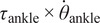	49 W	79%	42%

#### 6.2.2. Self-selected speed test

The condition that was completed in the shortest time was the second assistance torque condition, resulting in a 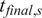 of 4 min 20 s.

The results are shown in Figure 11(b). Running with assistance torques increased the metabolic cost of running by 2.7%, while running speed increased by 11.4%, resulting in an overall decrease in the cost of transport by 7.7% as compared to running in normal shoes. The 2.7% increase in metabolic cost when running with assistance might seem to suggest that the subject performed worse in this test. However, since the subject also ran 11.4% faster in the same trial, this resulted in a combined improvement in cost of transport of 7.7%. This 7.7% reduction in the cost of transport is very promising. A previous running assistance device tested its event detection algorithm outdoors (Kim et al., 2019), but ours is the first device that has been shown to improve the cost of transport of running outside of the lab. Providing running assistance outdoors is more challenging as the exoskeleton needs to be able to provide stable and reliable torque assistance despite the higher variance in the subject’s gait due to speed changes, uneven ground, turning, and other outdoor variability. Since the cost of transport of running is almost the same across various speeds (Kram and Taylor, 1990), the percentage metabolic improvements in other studies that were conducted at fixed speeds would be equivalent to their percentage cost of transport improvements. This allows us to make direct comparisons between the results. The 7.7% reduction in cost of transport is on par with the current best-performing device tested on a treadmill which produced an 8.0% reduction in metabolic cost with a passive hip exoskeleton (Nasiri et al., 2018). This is very promising, given the additional challenges of providing running assistance outdoors.

Running in the zero torque condition decreased the metabolic cost of running by 7.3%, while running speed decreased by 16.0%, resulting in an overall increase in the cost of transport of 10.3% as compared to running in normal shoes. The 10.3% increase in the cost of transport is much lower than the 34.0% increase seen in the fixed speed test. Since the subjects stayed in aerobic respiration during the self-selected speed tests by lowering their running speed, we believe that the results from this test are more reliable. The 10.3% increase in the cost of transport is much lower than the 17% estimate based on the device mass distribution. We believe that this is because added mass has a lower impact on metabolic cost at lower speeds (Browning et al., 2007; Coifman et al., 2023) and the 17% estimate was based on tests performed at higher running speeds than the subject’s self-selected speed in the zero torque condition.

## 7. Future steps toward improving rope lifespan

We designed an extremely high-power application of twisted string actuators. Our device had a peak power level that was 7 times the peak power level of the next highest power twisted string device (Seong et al., 2020). We were met with challenges regarding the lifespan of our ropes, but the very promising preliminary results suggest that it is worth looking into methods to extend rope lifespan in high-torque twisted-string actuator applications. In this section, we look into our suggestions for how best to approach this challenge.

We can reduce the actuation load transmitted through each pair of twisted strings by increasing the number of twisted string pairs. The mechanism to parallelize motor torque across multiple sets of twisted strings should consist of only a few lightweight shafts, gears, and supporting hardware and have a minimal impact on the user’s metabolic cost of running.

We also found that the ropes had a significantly longer lifespan if used for multiple, shorter durations at a time. Some applications might take advantage of this behavior to extend their rope lifespan.

When analyzing the mechanisms of friction wear on the ropes, we identified two modes of wear that we would like to address. Firstly, the contact patch between the two ropes shifts as the ropes twist and untwist, causing inter-rope friction. Second, when the ropes are twisted, the fibers apply a squeezing force toward the twist axis (Figure 12). This squeezing force makes fibers move toward the twist axis at the midpoint of the rope bundle. This effect causes the cross section of the rope bundle to look more like a unified single circle, rather than separate ovals corresponding to the individual ropes. For a two-rope bundle, each rope ends up having a semicircular cross section area. When the ropes untwist, the fibers return to having a more even distribution of tension and return to their circular cross sectional areas. The fibers continue to rearrange themselves each time the rope twists and untwists, resulting in high intra-rope friction heating and wear.

Adjustments to the kinetostatic model of twisted strings should be made to account for the way the fibers in a rope bundle get rearranged into a single circle. This results in large changes to the effective rope radius which affects the modeling of force transfer through the twisted strings.

The high amount of inter-rope and intra-rope friction suggests that fiber weave might affect rope lifespan. The ropes used in the current exoskeleton had braided fibers. When a braided rope is twisted, only 50% of the fibers are woven in the twist direction and experience the torsional component of the tensile force. A rope made up of fibers that run parallel to each other might experience a more even distribution of loading and friction heating across the fibers.

The lifespan of twisted string actuators has been shown to decrease with increasing load forces, number of strings used, and stroke percentage (Usman et al., 2017). Such tests are very informative, but have only been performed for a range of forces 5 times smaller than what our device is experiencing. Tests should be performed to study the life cycle of twisted strings for higher loads and across more variables, such as stroke velocity, string radius, fiber weave pattern, and lubrication.

In Figure 13, we present our device’s actuation force 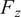 and stroke percentage 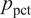 to provide a data point for future device development. 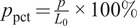 with 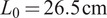 being the initial rope length.

## 8. Conclusion

This paper presents the design and testing of the first twisted string actuated exoskeleton that assists running. We focused on creating a lightweight design, through careful modeling of our transmission to select minimal mass components, as well as through mass-efficient design and placement of our components. Experimental results show that we are able to apply large assistance torques of 43 Nm kg 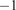 . We have learned that it is possible to obtain large metabolic cost savings during running with high-power twisted-string-actuated ankle exoskeletons. Preliminary tests on one subject show that we can reduce the metabolic cost of fixed speed running by 10.8% and reduce the cost of transport of running outdoors at a self-selected speed by 7.7%. We have also learned that the limiting factor for high-power twisted string applications is rope durability. However, the promising metabolic results indicate that efforts to improve rope lifespan during high-torque twisted-string applications could unlock improvements for powered, portable robotics. Future work should be done to study methods of increasing twisted rope lifespan at high torque levels. Once device durability is improved, we should repeat our study investigating metabolic rate impact on more subjects and across a wider population to understand if the device is capable of providing generalizable benefits across groups of different ages and mobility levels.

## Supporting information

Tan and Collins supplementary material Tan and Collins supplementary material

## Data Availability

The data that support the findings of this study are available from the corresponding author, S.H.C. upon reasonable request.

## Supplementary material

The supplementary material for this article can be found at http://doi.org/10.1017/wtc.2025.10010.
