# Supplementary material for: Design of an ankle exoskeleton with twisted string actuation for running assistance
Source: Wearable Technol. 2025 Jul 22;6:e34. doi: 10.1017/wtc.2025.10010 (PMC12304788; doi:10.1017/wtc.2025.10010)
Supplement: Tan and Collins supplementary material [file S2631717625100108sup001.pdf]

Supplementary Figures

Design of an Ankle Exoskeleton with Twisted String Actuation for Running Assistance

Guan Rong Tan[1] and Steven H. Collins[1*]

[1]Department of Mechanical Engineering, Stanford University, Stanford, CA 94305, USA.
*Corresponding author E-mail: stevecollins@stanford.edu

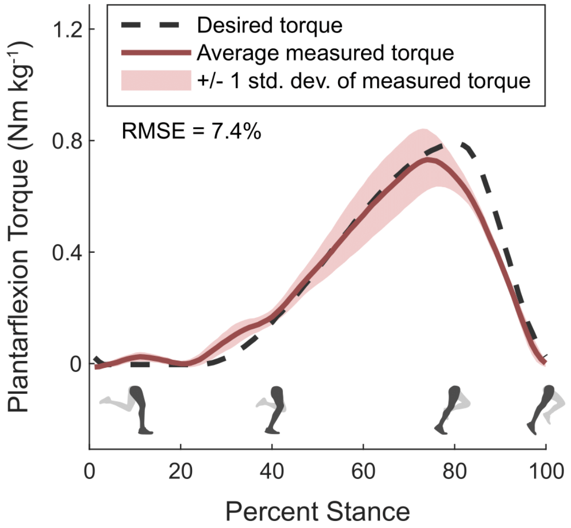

Figure S1. Ankle torque tracking during the final 20 strides prior to transmission failure. The reduction in peak torque and increase in torque tracking error during the push-off phase of stance (80% to 100% of stance) indicate that transmission wear led to higher transmission losses, motor saturation, and lower power production at the ankle joint.
